# Functionalized Quantum Dot‐Based Portable Sensor for Dual Detection of Stress Biomarkers in Blood Serum and Saliva

**DOI:** 10.1002/jbio.70268

**Published:** 2026-04-19

**Authors:** Anusha Kishore, Arun Mathew Varughese, Bernhard Roth, Carsten Zeilinger

**Affiliations:** ^1^ BMWZ (Centre of Biomolecular Drug Research) Gottfried Wilhelm Leibniz University Hannover Hannover Germany; ^2^ Hannover Centre for Optical Technologies Gottfried Wilhelm Leibniz University Hannover Hannover Germany; ^3^ Cluster of Excellence PhoenixD Gottfried Wilhelm Leibniz University Hannover Hannover Germany

**Keywords:** biochip, dual biomarkers detection, Hsp70, Hsp90, PMT‐scanner, point‐of‐care tests, portable sensor, quantum dots, stress biomarkers, TIQD imager

## Abstract

This work reports on the simultaneous detection of cellular stress biomarkers in blood and saliva, using a portable sensor. We expanded the application of our optical measurement system, the Transilluminated Quantum Dot (TIQD) imager, paired with a low‐cost biochip for minimally invasive biomolecule detection. Streptavidin functionalized quantum dots (Sav‐QDs) were conjugated with biotinylated antibodies (Ab) against stress biomarkers heat shock protein (Hsp). The presence of biomarkers in biological samples was indicated by the fluorescence masking of Sav‐QDs, based on their binding to the Abs conjugated to Sav‐QDs, resulting in reduced fluorescence. The decrease in fluorescence directly correlates with biomarker concentration. We have successfully detected Hsp90 and Hsp70 spiked in artificial blood serum. The limit of detection (LoD) was around 50 nM for Hsp90 and 96 nM for Hsp70. Our portable sensor addresses the need for reliable point‐of‐care (PoC) stress diagnosis by enabling robust, multiple biomarkers detection in diverse biological samples.

## Graphical abstract

The graphical abstract illustrates the principle of fluorescence masking: increasing concentrations of stress biomarkers lead to progressive masking of quantum dots' fluorescence, enabling their quantitative detection on a biochip with a benchtop imager.

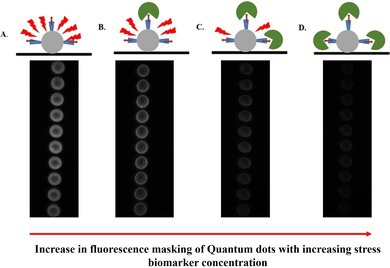

## 1 Introduction

Stress has been consistently linked to numerous physical and mental health issues and has been a significant factor in increased mortality rates. A 2022 Gallup survey found that 41% of adults globally experience high levels of stress. Stress disrupts molecular balance, leading to compromised immunity, increased inflammation, hormonal imbalances, and mental health disorders, affecting approximately 16% of the global population with stress‐induced hormonal issues, such as thyroid dysfunction. Chronic occupational stress affects 23% of workers in developed nations (WHO 2019), and undiagnosed burnout is a major concern for athletes. In sports, understanding recovery mechanisms and preventing overuse injuries is paramount. Blood‐based biomarkers play a critical role in assessing an athlete's fitness status after training or rehabilitation. They offer high specificity and precision. These biomarkers are released in response to physiological stress, such as during and after exercises and recovery periods [1]. At rest, biomarker levels return to baseline, indicating restored homeostasis and recovery [2, 3]. Blood‐based biomarkers are of great interest because of their high specificity, reproducibility, precision, and minimal interference during testing [4, 5, 6]. Hsp90 and Hsp70 are notable cellular stress biomarkers that reflect the degree of stress, systemic recovery, and long‐term effects of stress [7, 8]. Blood‐borne biomarkers of stress and recovery include cytokines, chaperones, enzymes, neurotransmitters, co‐factors, and inflammatory markers [9, 10]. Salivary biomarkers are increasingly being investigated as they reflect qualitative and quantitative changes in plasma components noninvasively [9]. Because of its simple, noninvasive sampling collection procedures, saliva is favored for PoC testing. By analyzing biomarkers in saliva samples, stress and stress‐related conditions can be effectively monitored. For example, the presence of Hsp90 in saliva and salivary glands indicates salivary gland cancer [11, 12, 13]. Multiplex analysis of biomarkers provides reliable and sufficient diagnostic information, especially in immunoassays, although there is a clear need for rapid, user‐friendly, and cost‐effective multiplex assay tests [14].

Current methods for stress monitoring predominantly require expensive laboratory tests or rapid PoC tests that are limited to single biomarker assessments. This results in a lack of comprehensive and cost‐effective solutions for monitoring stress in the field. Existing techniques often suffer from limited sensitivity, providing only approximate concentrations or binary (yes/no) results. Multi‐biomarker stress analysis typically requires laboratory tests, which, while sensitive, are time‐consuming, costly and require specialized personnel to perform the test. In the laboratory setting, common screening and monitoring techniques include mass spectrometry and highly sensitive fluorescent immunoassays, such as enzyme‐linked immunosorbent assay (ELISA) and surface plasmon resonance (SPR) [15]. Commercially available portable sensors for electrolytes and biomarkers illustrate the current technological landscape. However, these solutions still require significant development, particularly with regard to the quantification of biochemical markers by wearable sensors to accurately reflect physiological stress, recovery, and fitness [16]. Current optical wearable sensors face challenges in detecting subtle biomarker fluctuations over short intervals for continuous monitoring and predominantly provide binary results. In addition, biocompatibility with the skin continues to be a significant barrier to the further development of wearable sensor technology [17].

Currently, nanoparticles such as carbon dots, graphene, metallic nanoparticles, and semiconductor nanoparticles are widely used in the development of PoC biosensors [18]. Semiconductor nanoparticles, particularly QDs, are often used as dyes in biosensing due to their inherent fluorescence or photoluminescence. QDs are particularly useful in droplet‐based multiplex assays for high sensitivity and parallel analysis of small samples in the picolitre range [19]. As semiconducting nanocrystals, QDs consist of heavy metals or inorganic elements such as cadmium (Cd), selenium (Se), zinc oxide (ZnO), and silicon (Si), with dimensions ranging from 2 to 10 nm. They are often coated with biocompatible polymers like polyethylene glycol, which provides free carboxyl (—COOH) or amine (NH_2_) groups, rendering the QDs water‐soluble and suitable for conjugation with biomolecules [20, 21, 22]. Bioconjugation can occur through either covalent or non‐covalent interactions. In covalent binding, functional groups such as carboxyl (—COOH) on QDs form stable bonds with functional groups on biomolecules. In contrast, non‐covalent binding relies on electrostatic interactions or high‐affinity secondary interactions facilitated by molecular adapters, such as the avidin‐biotin system [22, 23]. Streptavidin, conjugated to QDs, that is, Sav‐QD, specifically interacts with biotin. This system allows the attachment of a wide variety of biomolecular ligands via biotin, facilitating the formation of QD‐antibody complexes capable of detecting target biomarkers in diverse test samples [23]. QDs are widely used as biosensing probes in PoC bioassays and biosensors. Of particularly interest are QD‐based fluorescence resonance energy transfer (FRET) bioassays which act as donors for organic dyes, dye‐labeled proteins, or other nanoparticles. These assays require no additional washing steps and are known for their high sensitivity and selectivity, making them suitable for detecting infectious agents, such as influenza virus antigens [24, 25, 26, 27, 28]. Fluorescence‐based PoC assays have revolutionized PoC diagnostics [11, 12]. The concept of fluorescence masking of Sav‐QDs on a biochip has been extended to study protein interactions in the presence of an inhibitor, such as the interaction between Hsp90 and thyroid hormone receptor beta (TRβ) in the presence of triiodothyronine (T3). This principle has also been used to detect disease‐causing antigens, such as the spike protein, in the presence of its receptor, the Angiotensin‐converting enzyme 2 (ACE2) [29, 30].

Recently, there has been a growing demand for research focused on the development of smartphone‐based optical readout systems for PoC and QD‐based bioassays [31, 32, 33, 34]. Also, smartphones are increasingly used for this purpose, enabling modular and compact systems for multiplexed biosensing which were not accessible before [35]. The cameras in smartphones are capable of measuring various optical phenomena, including fluorescence, absorbance, surface plasmon resonance, as well as detecting Raman signals, thus making them valuable diagnostic tools, especially in resource‐constrained settings [36]. The capability to detect fluorescence changes in QDs has become more accessible with advancements in smartphone technology and portable spectrophotometers [37].

The aim of this work was to develop and optimize the processing steps for a QD‐based biochip, which allows testing of different stress biomarkers in different biological samples. The biochip was optimized for direct detection of stress biomarkers at the end‐user site. This work utilizes a low‐cost benchtop TIQD imager, specifically designed to detect fluorescence masking of QDs by bulky proteins and was calibrated by purified Hsp90 through protein‐antibody interactions in a standard buffer solution [38, 39]. The system is smaller and more sensitive than traditional, more expensive photomultiplier tube (PMT) scanners, offering even lower detection limits. The TIQD imager, designed for PoC applications, has initially been used for the detection of pure Hsp90 [40, 41, 42]. In this study, it was developed further to extend its use for the detection of the stress biomarkers Hsp90 and Hsp70 in artificial blood serum and saliva. The biochip, as an innovative sensor, together with the TIQD imager, offers a more rapid and cost‐effective testing alternative compared to traditional ELISA or PMT‐based methods, paving the way for rapid PoC stress diagnostics with significant applications in the sports sector. In the future, integration with mobile phone technology could provide a low cost, reliable self‐monitoring and diagnostic tool.

## 2 Experimental Procedure

The initial phase of this research by Kishore et al. involved developing a biochip printed with Sav‐QDs to detect purified Hsp90 via a portable benchtop optical device, the TIQD imager [40, 41, 42]. In this work, the biochip was taken to the next level and optimized to detect two cellular stress proteins (Hsp90 and Hsp70) concurrently in blood serum and saliva. The shelf life of the biochip was also assessed under standard storage conditions. The detection range of the overall system was adjusted to the clinically viable range at picomolar (pM) levels. Data from measurements with the standard PMT‐scanner were used as a reference for the results from the TIQD imager. The binding of stress proteins to the printed Sav‐QDs was confirmed using cross‐reactivity tests.

### 2.1 Materials

The biotin‐conjugated antibodies used in this study were Biotin anti‐Hsp90 (Biotin Rabbit Polyclonal to Hsp90, abx445722, Abbexa, Netherlands), Biotin anti‐Hsp70 (Biotin Rabbit Polyclonal to Hsp70, abx445606, Abbexa, Netherlands), and ACE2 Ab (Biotin, Rabbit Polyclonal to ACE2, BIOZOL, Germany). The Anti‐His antibody used was a mouse monoclonal to His‐tag (SAB1305538, Sigma Aldrich, Germany). ACE2 (ACROBiosystems, Germany) of molecular weight 87.2 kDa is used in the experiment. Artificial blood serum (Human Male AB, H4522, Sigma Aldrich, Germany) and artificial saliva (Human, BZ323, Bio Chemazone, Canada) were also utilized. The storage solution was obtained from Candor (Germany). The PBS buffer was prepared in the laboratory with 137 mM NaCl, 2.7 mM KCl, 10 mM Na_2_HPO_4_, and 1.8 mM KH_2_PO_4_, maintaining a pH of 7.4.

Human Hsp90 alpha (stock concentration 3 mg/mL) was recombinantly synthesized and purified according to the methods described by Schax et al. and Yue et al. [43, 44]. Human Hsp70 (stock concentration 3 mg/mL) was synthesized and purified as described in the work by Mohammadi et al. [45].

### 2.2 Methodology

The biochip was printed using a mixture of 50 nM Sav‐QD and 500 nM biotinylated Ab (QD + Ab). The Abs used were specific for either Hsp90 or Hsp70. The fabrication of the biochip and the reference measurements with the PMT‐scanner were conducted as described in the initial study. A detailed description of the protocols and the methodology can be found in Kishore et al. [41].

**FIGURE 1:**
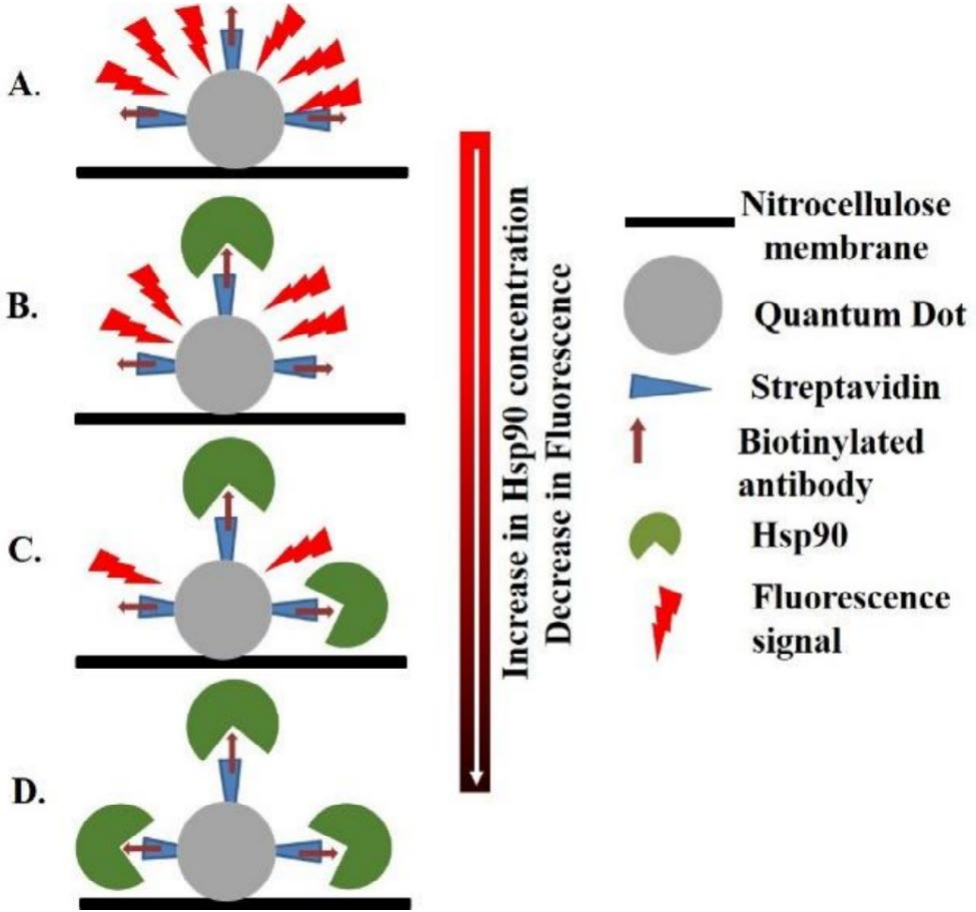
Schematic overview of the biochip and fluorescence masking concept. (A) QD + Ab solution was printed on a nitrocellulose pad of the biochip. (B) Addition of low concentration of Hsp90. (C) Addition of medium concentration of Hsp90. (D) Addition of high concentration of Hsp90 that completely blocks fluorescence signal [42].

The QD + Ab solution was initially spotted onto a nitrocellulose membrane, establishing a foundational layer for subsequent interactions. As shown in Figure 1 upon introduction of a low concentration of Hsp90, binding to the QD + Ab occurs, resulting in a partial masking of the fluorescence signal. This initial interaction indicated the beginning of fluorescence masking. With the addition of a medium concentration of Hsp90, further binding occurred, leading to a more pronounced masking of the fluorescence signal, demonstrating the concentration‐dependent nature of the fluorescence masking effect. Finally, the application of a high concentration of Hsp90 leads to saturation binding, completely masking the fluorescence signal of the QDs. This step exemplifies the maximal fluorescence masking when target stress biomarker concentrations were high.

**FIGURE 2:**
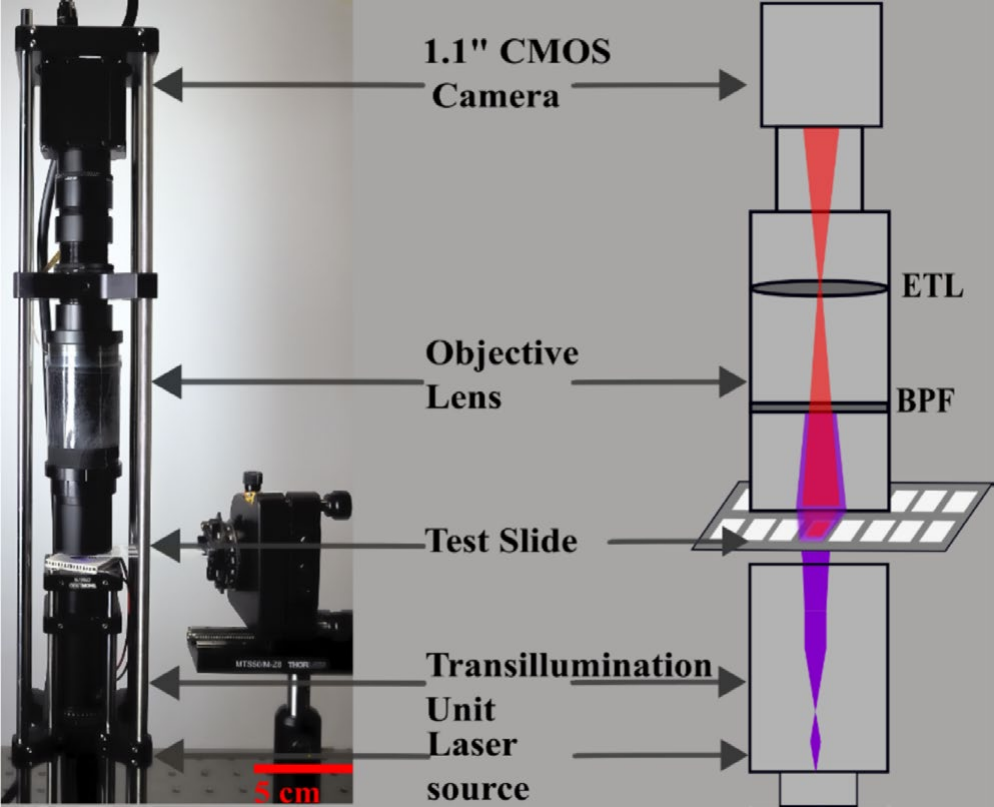
(Left) Image of the portable TIQD imager (volume 10 × 10 × 30 cm), mounted on top of the biochip with Hsp90/Hsp70. (Right) Schematic layout of the imager exciting QD + Ab spots with a 405 nm laser producing fluorescence with a maximum around 655 nm. BPF, band‐pass filter blocking of the excitation light; ETL, electrically tunable lens used for auto‐focusing of the test biochip.

The portable TIQD imager, illustrated in Figure 2, is simple and relies on fluorescence detection. Sav‐QDs exhibit a broad absorption spectrum with a notable molar absorption coefficient of approximately 1 080 000 cm^−1^ M^−1^ in the ultraviolet (UV) region, which gradually decreases toward the visible (VIS) spectrum [46]. Maximal emission is found at a wavelength of 655 nm. For excitation, a 405 nm diode laser (RLDE405M‐20‐5, Roithner Lasertechnik GmbH, Vienna, Austria) with an output power of 20 mW was utilized. This ensures efficient separation of excitation and emission wavelengths and, thus, enhances spatial resolution. A trans‐illumination configuration was employed to excite the Sav‐QDs bound onto the nitrocellulose pad on the biochip. Optimal positioning of the excitation source close to the test sample minimized optical losses [47]. To achieve a uniform illumination profile across the entire field of view, the elliptical beam profile of the laser beam was reshaped using a combination of optical elements: a plano‐convex lens (LA1951), a negative meniscus lens (LF1822), and a square diffuser (ED1‐S50), all from Thorlabs GmbH, Munich, Germany.

The field of view is 5 × 5 mm, which allows to image a single pad on the nitrocellulose‐coated glass slides (2UNY2GW00600616G, Sartorius AG, Göttingen, Germany). The optical system utilized for imaging includes a 1.1″ IMX304 CMOS grayscale camera (U3‐3200SE‐M‐GL, IDS Imaging Development Systems GmbH, Germany). Zemax ray‐tracing simulations were used to minimize the working distance between the imaging lens and the target sample and to enhance the collection efficiency of emitted fluorescence. For this, a positive meniscus lens (LE1234, Thorlabs GmbH, Munich, Germany) is integrated into the objective assembly, reducing the working distance to approximately 10 mm. This lens configuration corrects for spherical aberrations while maintaining high image resolution. Using the simulations, the optical system was optimized to achieve a numerical aperture (NA) of approximately 0.7, again to increase collection efficiency. An electrically tunable lens (EL‐12‐30‐TC‐VIS‐16D, Optotune, Switzerland) is incorporated to enable automated focusing via a focus stacking technique, ensuring optimal image quality throughout the measurement process. The values calculated from a series of images (5–10) taken at different focal lengths are averaged to mitigate errors arising from sample surface unevenness. To prevent residual excitation light from interfering with the fluorescence signal, a narrow band‐pass filter (FBH650‐10, Thorlabs GmbH, Munich, Germany) is placed in the optical path. This filter selectively transmits the fluorescence emitted by the QDs while effectively blocking the excitation wavelength of the laser.

### 2.3 Biochip Usage for Detecting Stress Biomarkers in Blood Serum and Saliva

Artificial blood serum was spiked with varying concentrations of Hsp90 and Hsp70. These concentrations were prepared in PBS buffer and thoroughly mixed with the serum. The experimental procedure for detecting Hsp70 was identical to that used for Hsp90. Briefly, a dilution series of the target protein (Hsp90 or Hsp70) was first prepared in PBS buffer. This prepared dilution was then used to spike the artificial blood serum, creating the final sample solution that was applied to the biochip for incubation and analysis. A volume of 100 μL of the spiked blood serum sample was pipetted onto the surface of the biochip pad and incubated in an incubation chamber at 4°C for 1 h. Following incubation, the biochips were analyzed using both the PMT‐scanner and the TIQD imager to quantify fluorescence masking. The data were used to determine the limit of detection (LoD) for each method.

Also, artificial saliva was similarly spiked with varying concentrations of Hsp90, following the same procedure. The spiked saliva was then applied to the biochip and incubated for 15 min at 4°C in dark conditions. Subsequently, the biochip results were analyzed. The shorter incubation time for saliva was empirically determined to minimize the degradative effects of salivary enzymes on the nitrocellulose coating and the QDs. The effect of artificial saliva spiked with Hsp90 on printed Sav‐QDs after 1 h of incubation was also observed. This was done to determine the optimum incubation time of the saliva sample on the biochip.

The control values taken as “0 nM” Hsp90 or Hsp70 represent the signal from artificial blood serum or saliva samples without spiking with Hsp90 or Hsp70.

### 2.4 Imaging of the Biochip

Area‐based gridding was implemented for each image captured by the TIQD imager, with the grids aligned to the number of spots on each pad. Within these grids, pixel segmentation was conducted to distinguish between foreground and background pixels. The intensity of each QD spot was calculated by subtracting the average background intensity from the average foreground intensity. These spot measurements were averaged to yield a single representative value for each pad. The fluorescence signals were then evaluated for each pad with (*F*) and without (*F*_0_) the presence of Hsp90 or Hsp70. The reference measurement (*F*_0_) was used to correlate with the known concentrations of Hsp90 for the corresponding pads. *F*_0_ − *F* represents the absolute signal difference (masking effect) and is used to show the raw data. *F*_0_/*F* is the signal ratio used for generating the standard curve and calculating the LoD, as it normalizes for variations in initial spot intensity and is the standard parameter for such calculations in masking (fluorescence quenching) based assays.

### 2.5 Dual Biomarkers Detection of Stress Biomarkers

Biochips for dual biomarkers detection were fabricated by printing the QD + Ab solution onto each pad, with Hsp70 Abs printed in the first column and Hsp90 Abs printed in the second column. The blood serum was spiked with a mixture of Hsp70 and Hsp90 proteins (collectively termed Hsps). Hsp90 and Hsp70 were mixed in a 1:1 M ratio for each concentration point (500 nM Hsp90 + 500 nM Hsp70). Varying concentrations of Hsp70 and Hsp90 were prepared in PBS buffer and subsequently combined with the blood serum. After incubation with the Hsps, the biochips were subjected to analysis through both the PMT‐scanner and the TIQD imager.

### 2.6 Lowering the Detection Range

In a previous study conducted by Fan et al., the concept of fluorescence masking was utilized to investigate protein–protein interactions between Hsp90 and thyroid hormone receptor beta (TRb). Initially, fluorescence masking was induced by Hsp90, with an additional masking effect contributed by TRb [29]. Building on this foundation, it was hypothesized that the addition of a secondary layer of large proteins, such as a secondary Ab, on top of Hsp90 molecules attached to QD + Ab complexes on the biochip could enhance the overall fluorescence masking of the QDs. To test this hypothesis, biochips printed with QD + Ab were incubated with varying concentrations (250–0.25 nM) of His‐tagged Hsp90 for 1 h at 4°C. Subsequently, 250 nM of Anti‐His secondary Ab was added to each Hsp90 concentration and incubated for an additional 1 h at 4°C. The biochip was then analyzed to assess the effects of this additional protein layer on fluorescence masking.

### 2.7 Shelf‐Life Test and Evaluation of Cross‐Reactivity

The biochip provides a dried and stable state for antibodies bound to Sav‐QDs, which improves their shelf life under standard storage conditions of 4°C in a dry and dark environment. Additionally, the use of a storage buffer further enhances the shelf life of the biochip without compromising its functionality. The printed biochip was stored in a solution from Candor to assess its stability over time. The photostability of the printed Sav‐QDs and the activity of the biochip were measured over a period of 6 months. To assess whether unwanted binding occurs due to hydrophobic interactions, different concentrations of TP53 (Tumor suppressor protein) were used with Hsp90 and Hsp70 Abs. A concentration of 50 nM Sav‐QD was mixed with 500 nM of Hsp90 and Hsp70 Abs. The mixtures were printed separately in two columns on the biochip. Different concentrations of TP53 (500, 100, 50, 10, 5, 1, and 0 nM) were added to determine whether there was any concentration‐dependent fluorescence masking. TP53 was expressed as pEXP5CT_p53 plasmid construct and purified after expression from *E. coli* cells by Ni‐IMAC chromatography.

### 2.8 Interpretation of Fluorescence Masking

The fluorescence masking of Sav‐QDs is attributed to steric blocking by large proteins like Hsp90. To confirm the size/mass‐dependent effect, fluorescence masking by two different size proteins of the same concentrations was compared. The molecular size of Hsp90 dimer is 180 kDa and that of ACE2 is 85.9 kDa. 50 nM of Sav‐QD was printed on the biochip, and different concentrations of Hsp90 Ab and ACE2 Ab were added on two separate biochips. Different concentrations of Hsp90 and ACE2 were then added on the biochips with the respective antibodies. Fluorescence masking of Hsp90 and ACE2 was compared for each concentration.

## 3 Results and Discussion

### 3.1 Testing Stress Biomarkers in Blood Serum

**FIGURE 3:**
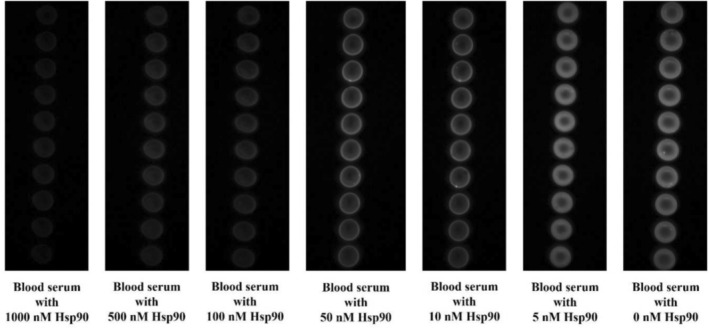
Images of the biochip, obtained using a TIQD imager, showing the fluorescence masking effect by varying Hsp90 concentrations on the fluorescence of the printed Sav‐QDs.

To investigate the extent of fluorescence masking of QD + Ab spots by Hsp90, a range of Hsp90 concentrations (1000, 500, 100, 50, 10, and 5 nM) was introduced to the biochip, as illustrated in Figure 3. The fluorescence measurement at 0 nM Hsp90, consisting solely of pure blood serum, served as the control. Results indicate an inverse relationship between Hsp90 concentration and fluorescence intensity, with higher concentrations resulting in increased fluorescence masking. The masking effect was quantified by calculating the difference in fluorescence intensity of the QD + Ab spots before (*F*_0_) and after (*F*) the addition of blood serum spiked with Hsp90. Importantly, the findings demonstrate that the presence of blood serum does not impede Hsp90's binding efficacy to the Abs, thereby confirming the reliability of using blood serum directly on the biochip for stress biomarker measurements. Each concentration was tested using 10 replicates of Sav‐QD spots per Hsp90 concentration, on a single biochip.

**FIGURE 4:**
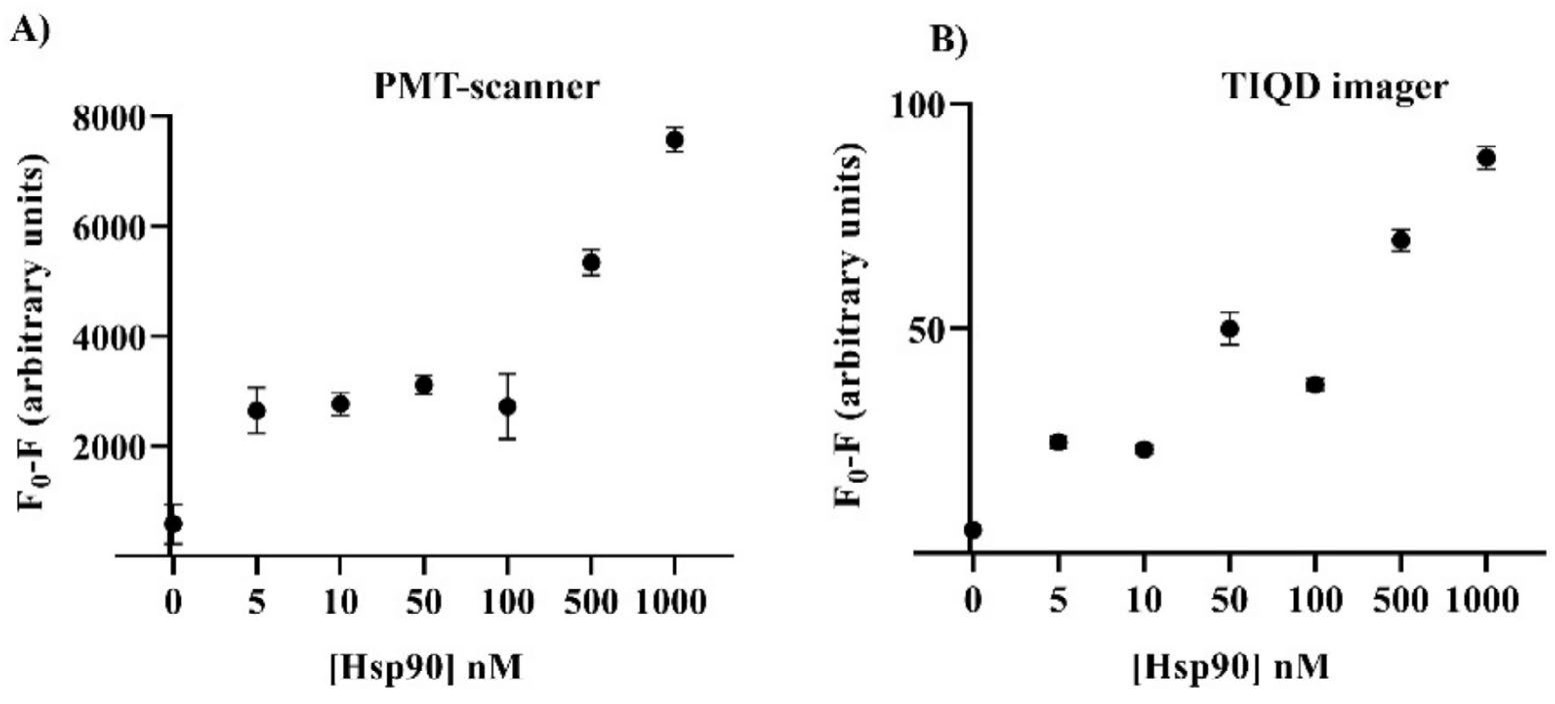
Fluorescence measurements made before and after incubation with blood serum spiked with Hsp90. Fluorescence masking is plotted against different concentrations of Hsp90. (A) Shows the fluorescence signal obtained by the PMT‐scanner and (B) by the TIQD imager, respectively. *F*_0_ is the fluorescence signal after the QD + Ab solution is printed on the biochip and *F* is the fluorescence signal from these spots after binding of Hsp90 to QD + Ab.

As shown in Figure 4A, the fluorescence masking measured by the PMT‐scanner decreases from 1000 to 100 nM Hsp90, after which the values show saturation for lower concentrations (100–5 nM). In Figure 4B, the results from the TIQD imager are presented, exhibiting a similar trend compared to those from the PMT‐scanner. The masking effect decreases till 100 nM and shows saturation with lower concentrations of Hsp90. The abnormal high value at 50 nM Hsp90 is likely attributable to aberrations in the printing process of the biochip.

**FIGURE 5:**
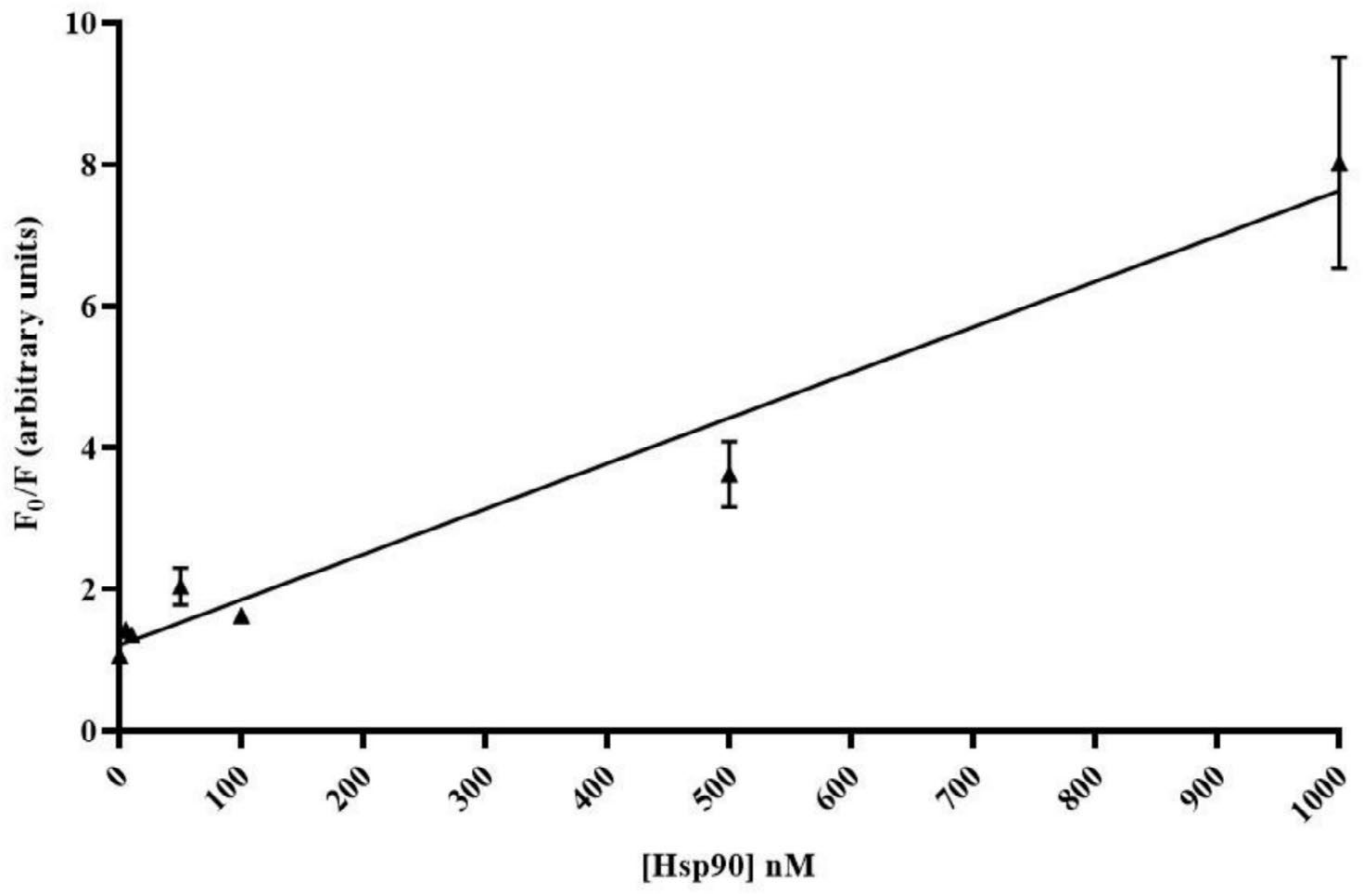
Linear regression plotted as *F*_0_/*F* versus Hsp90 concentration ranging from 1000 to 0 nM. The slope of the graph is *k* = 0.0064 nM^−1^ and *r* ^2^ = 0.9685.

For the TIQD imager as shown in Figure 5, a linear regression was plotted to determine the LoD for the system, utilizing the equation LoD=3σk, where *σ* represents the standard deviation of the control and *k* denotes the slope of the graph. The LoD calculated for the TIQD imager was 57.18 nM. At higher concentrations the significant fluorescence intensity decrease of the QD + Ab spots (as shown in Figure 3) causes increased measurement uncertainties.

**FIGURE 6:**
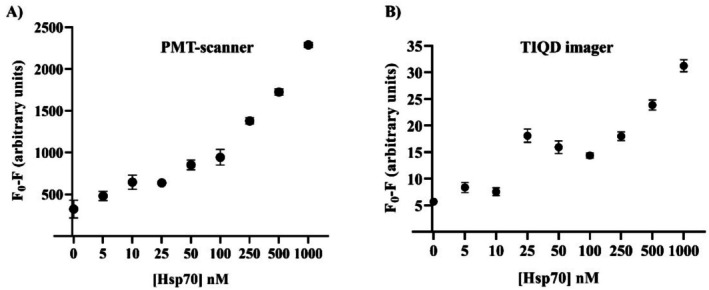
Fluorescence measurements made before and after incubation with blood serum spiked with Hsp70. Fluorescence masking is plotted against different concentrations of Hsp70. (A) Shows the fluorescence signal obtained by the PMT‐scanner and (B) by the TIQD imager, respectively. *F*_0_ is the fluorescence signal after the QD + Ab solution is printed on the biochip and *F* is the fluorescence signal from these spots after binding of Hsp70 to QD + Ab.

A second stress biomarker, Hsp70, was tested in the same manner as Hsp90, spiked in blood serum. Various concentrations of Hsp70 were added to the biochip to assess the fluorescence masking effect. Each concentration was tested using 10 replicates of Sav‐QD spots per Hsp70 concentration on a single biochip. As observed in Figure 6, Hsp70 demonstrated a similar trend to Hsp90. However, the fluorescence masking effect was more pronounced for Hsp90 compared to Hsp70 at equivalent concentrations.

**FIGURE 7:**
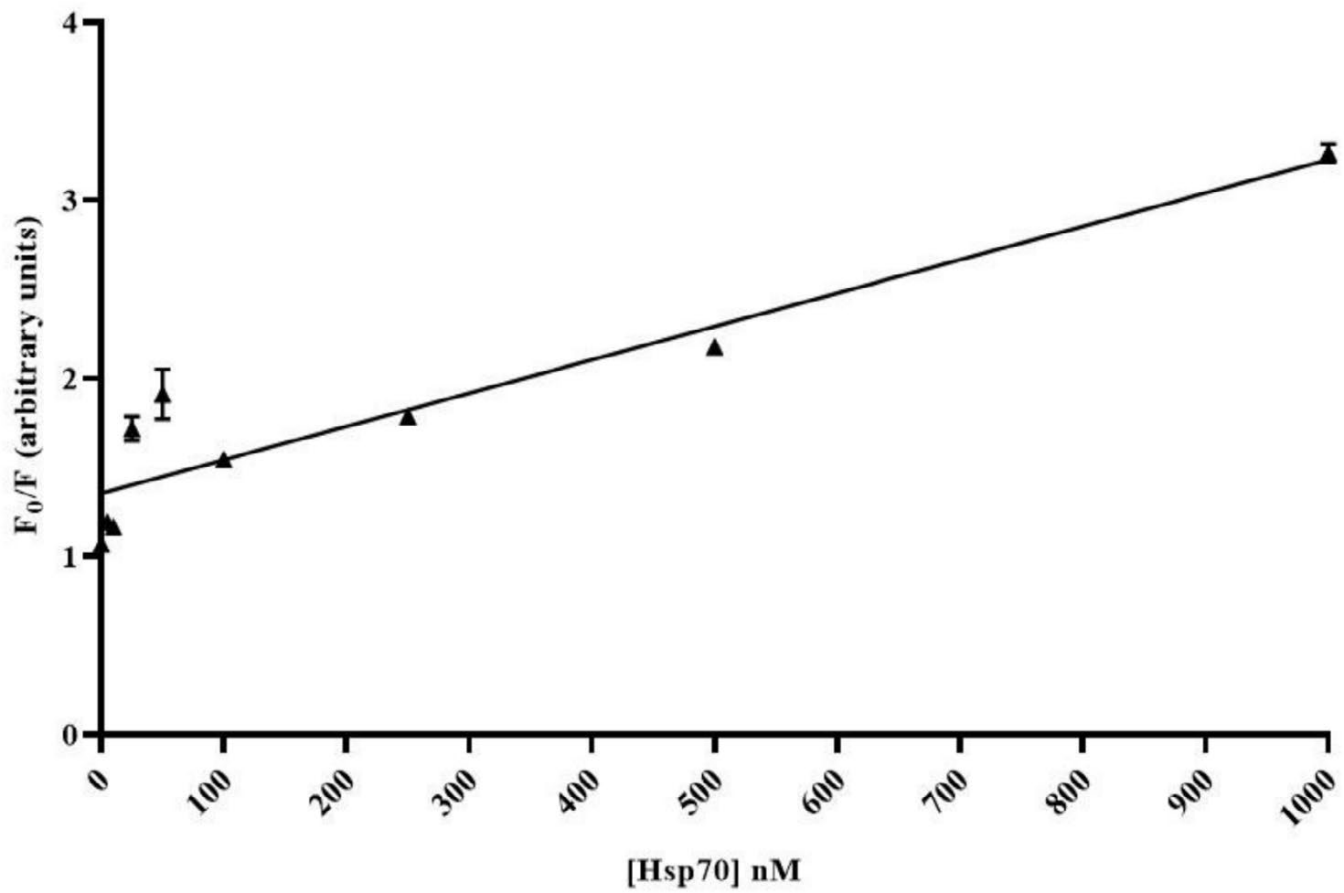
Linear regression plotted with *F*_0_/*F* versus Hsp70 concentration ranging from 1000 to 0 nM. *F*_0_ and *F* are fluorescence intensities of QD + Ab spots before and after adding Hsp70. The slope of the graph is *k* = 0.0018 nM^−1^ and *r* ^2^ = 0.8681.

As seen in Figure 6A, for the PMT‐scanner the fluorescence masking decreases from 1000 to 50 nM Hsp70; the values after 50 nM show saturation. In Figure 6B, the TIQD imager results show a similar trend; the abnormally high values at 50 and 25 nM are probably due to imaging aberrations. As per Figure 7, the LoD of the TIQD imager for Hsp70 was 96.102 nM, which is slightly higher than that for Hsp90.

The detection range refers to the concentration interval over which a measurable change in signal occurs (from ~1000 nM down to ~10–25 nM). The LoD is a statistically derived value (3*σ*/slope) indicating the lowest concentration that can be reliably distinguished from zero.

### 3.2 Hsp90 in Saliva

**FIGURE 8:**
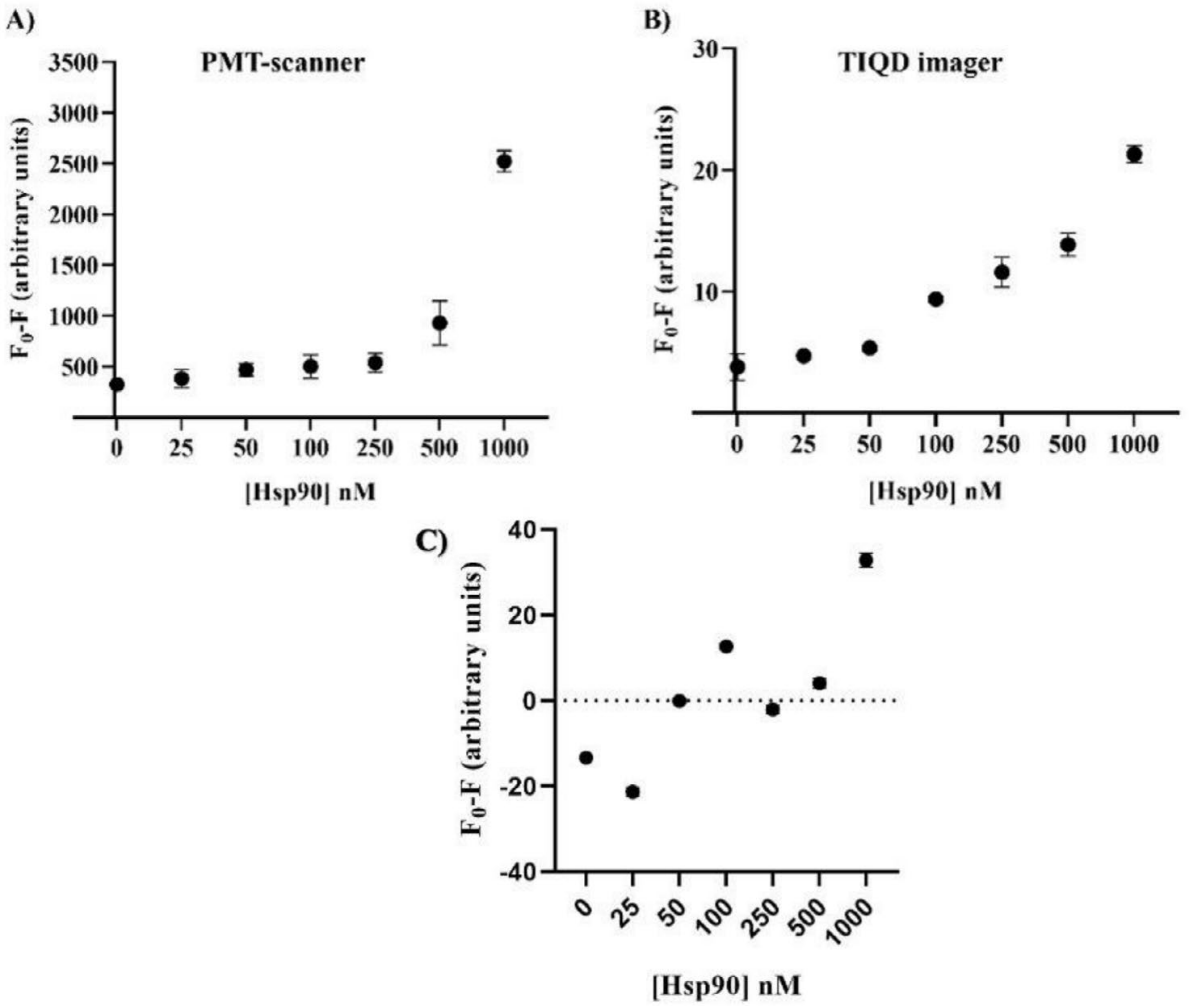
Fluorescence measurements made before and after incubation with artificial saliva spiked with Hsp90. Fluorescence masking is plotted against different concentrations of Hsp90. (A) Shows the fluorescence signal obtained by the PMT‐scanner and (B) by the TIQD imager, respectively. *F*_0_ is the fluorescence signal after the QD + Ab solution is printed on the biochip and *F* is the fluorescence signal from these spots after binding of Hsp90 to QD + Ab. (C) Fluorescence measurements were performed before and after 1 h of incubation with artificial saliva spiked with Hsp90. The fluorescence signal difference is plotted against different concentrations of Hsp90. The graph shows the fluorescence signal obtained by the TIQD imager. *F*_0_ is the fluorescence signal after QD + Ab solution is spotted on the biochip and *F* is the fluorescence signal from these spots after incubation with artificial saliva spiked with Hsp90.

To evaluate the compatibility of saliva as a biological sample with the biochip for stress biomarker testing, the fluorescence masking effect of QD + Ab spots for various concentrations of Hsp90 was assessed. A one‐time dilution of artificial saliva was performed using an extraction buffer, followed by spiking with Hsp90. This dilution was necessary to reduce the concentration of enzymatic components present in the saliva matrix, which could degrade the nitrocellulose surface of the biochip and impact the stability of the QDs over longer incubation periods, thereby compromising the assay's integrity and signal‐to‐noise ratio. The spiked saliva was then applied to the biochip, incubated for 15 min, and imaged. The incubation time was reduced from 1 h to 15 min to mitigate the effects of salivary enzymes on the nitrocellulose, QDs and optimize the results. This shorter time was sufficient for biomarker binding while preserving biochip functionality. Each concentration was tested using 10 replicates of Sav‐QD spots per Hsp90 concentration, on a single biochip. As shown in Figure 8A,B, for the PMT‐scanner, fluorescence masking decreases up to 250 nM Hsp90, after which it saturates. In contrast, the TIQD imager exhibited a decrease in fluorescence masking till 50 nM and saturation afterwards in lower concentrations of Hsp90 in artificial saliva. In the beginning, incubation of artificial saliva on the biochip was carried out for 1 h. As observed in Figure 8C, the fluorescence decreases only up to 500 nM Hsp90 concentration. After that, the values obtained with the TIQD imager are inconclusive. This indicates that the binding between Hsp90 and biotinylated Ab is not sufficient. This could be due to enzymes in saliva destroying the spiked Hsp90 or bleaching of the Sav‐QD and requires more systematic testing to understand the exact phenomenon in future.

The PMT‐based scanner demonstrated greater sensitivity at low light conditions (i.e., higher fluorescence masking at 1000–250 nM Hsp90) due to its high gain and low noise, compared to the CMOS sensor of the TIQD imager [40, 41].

### 3.3 Dual Detection of Stress Biomarkers in Blood Serum

**FIGURE 9:**
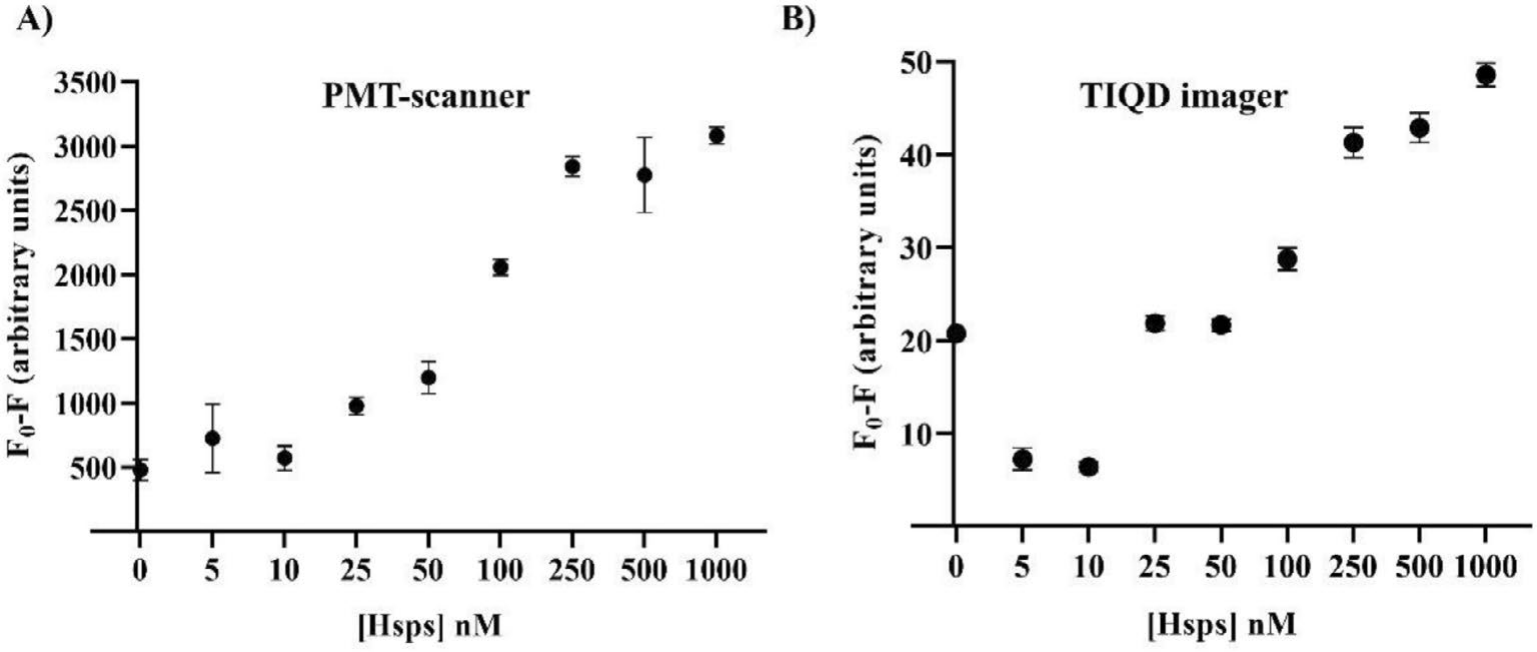
Fluorescence measurements made before and after incubation with artificial blood serum spiked with Hsps (Hsp90 + Hsp70). Fluorescence masking is plotted against different concentrations of Hsps. (A) Shows the fluorescence signal obtained by the PMT‐scanner and (B) by the TIQD imager, respectively. *F*_0_ is the fluorescence signal after the QD + Ab solution is printed on the biochip and *F* is the fluorescence signal from these spots after binding of Hsp90 to QD + Ab.

To assess the capability of simultaneous detection of multiple stress biomarkers, a mixture containing different concentrations of Hsp90 and Hsp70 in a 1:1 M ratio (collectively referred to as Hsps) was incubated on the biochip. Blood serum spiked with Hsps was utilized for this purpose. The dual detection of Hsp90 and Hsp70 was achieved using a spatial multiplexing approach. The same type of QDs (emission at 655 nm) was functionalized with either Hsp90 Ab or Hsp70 Ab and printed in separate, defined columns on the biochip. This design allows a single sample to be tested for both biomarkers simultaneously on one biochip's area (pad). The signal intensity from each specific antibody column provides the quantitative concentration for each individual biomarker, while the combined signal for Hsps offers a measure of the overall cellular stress level. Each concentration was tested using 10 replicates of Sav‐QD spots per Hsp90 and Hsp70 concentration, on a single biochip. As depicted in Figure 9, the fluorescence masking effect decreases from 1000 nM to around 10 nM for Hsps when using the PMT‐scanner and the TIQD imager. Upper saturation level is from 500 to 1000 nM and lower saturation level from below 25 nM Hsp90.

**FIGURE 10:**
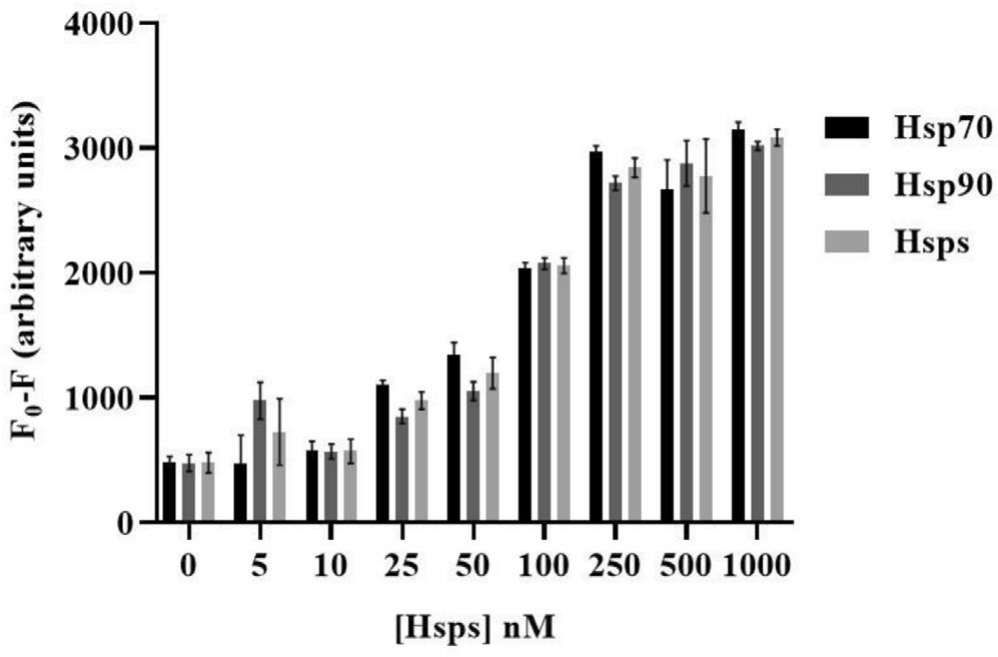
Comparing PMT‐scanner results of fluorescence masking by each Hsp70 and Hsp90 separately and together as Hsps.

In the case of the PMT‐scanner results for Hsps in Figure 10, it is observed that the masking effect at each concentration closely aligns with the values obtained for Hsp90 and Hsp70 when tested individually. Therefore, the combined results for Hsps can be effectively utilized for further evaluation of the LoD. The masking effect of different Hsps concentrations on QD + Ab spots coated with Hsp90 Ab and Hsp70 Ab separately is illustrated in Figure 10.

**FIGURE 11:**
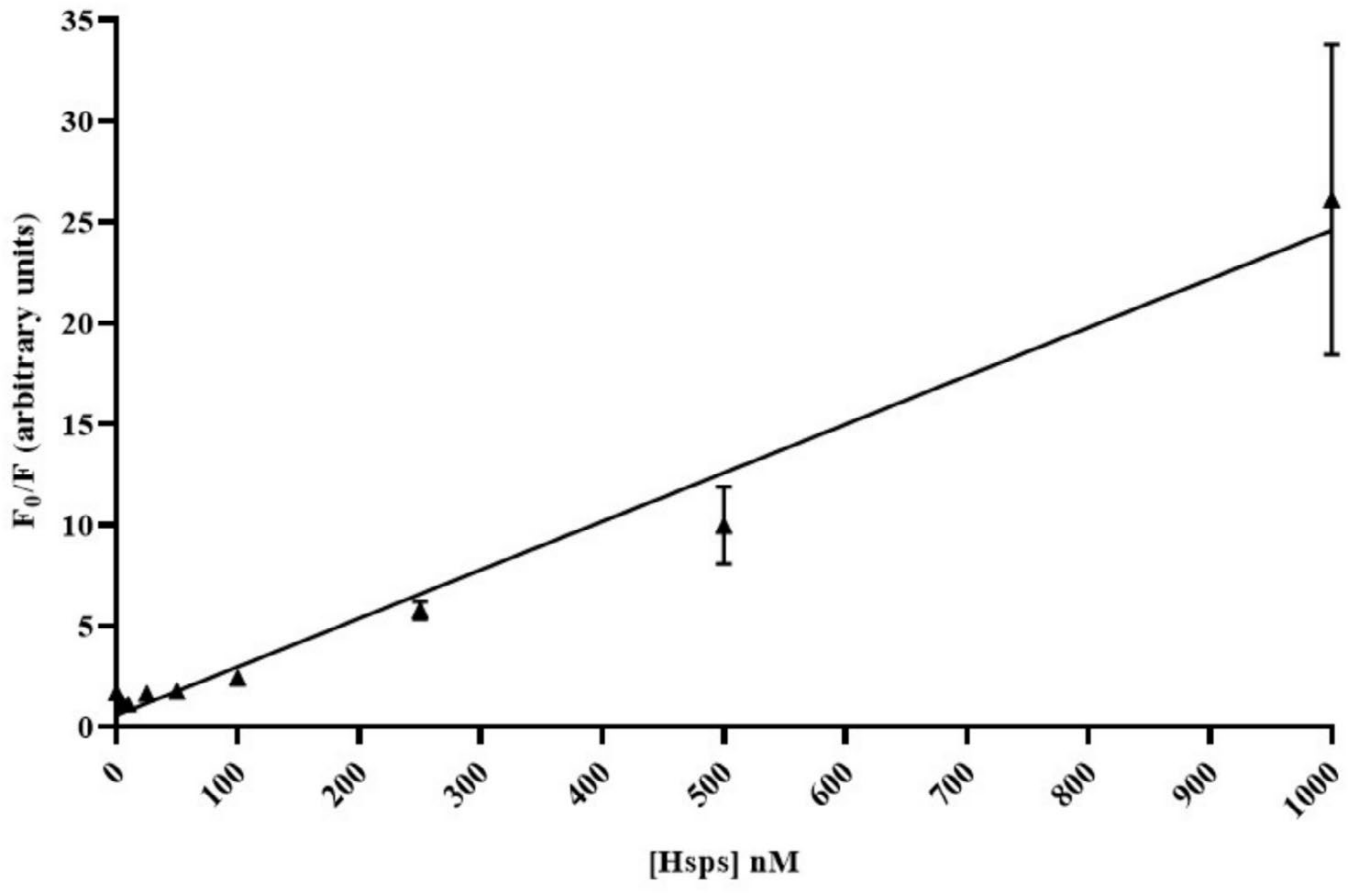
Linear regression was plotted for TIQD imager with *F*_0_/*F* versus Hsps concentration ranging from 1000 to 0 nM. *F*_0_ and *F* are fluorescence intensities of QD + Ab spots before and after adding Hsps. The slope of the graph is *k* = 0.024 nM^−1^ and *r* ^2^ = 0.9780.

The LoD for the TIQD system was calculated for Hsps collectively from Figure 11 and determined to be 26.23 nM. This value is lower than the LoD for either Hsp90 or Hsp70 in blood serum individually. As explained in section 3.1 at 1000 nM of Hsps, the significant fluorescence intensity decrease of the QD + Ab spots causes larger measurement uncertainties.

### 3.4 Lowering the Detection Range

**FIGURE 12:**
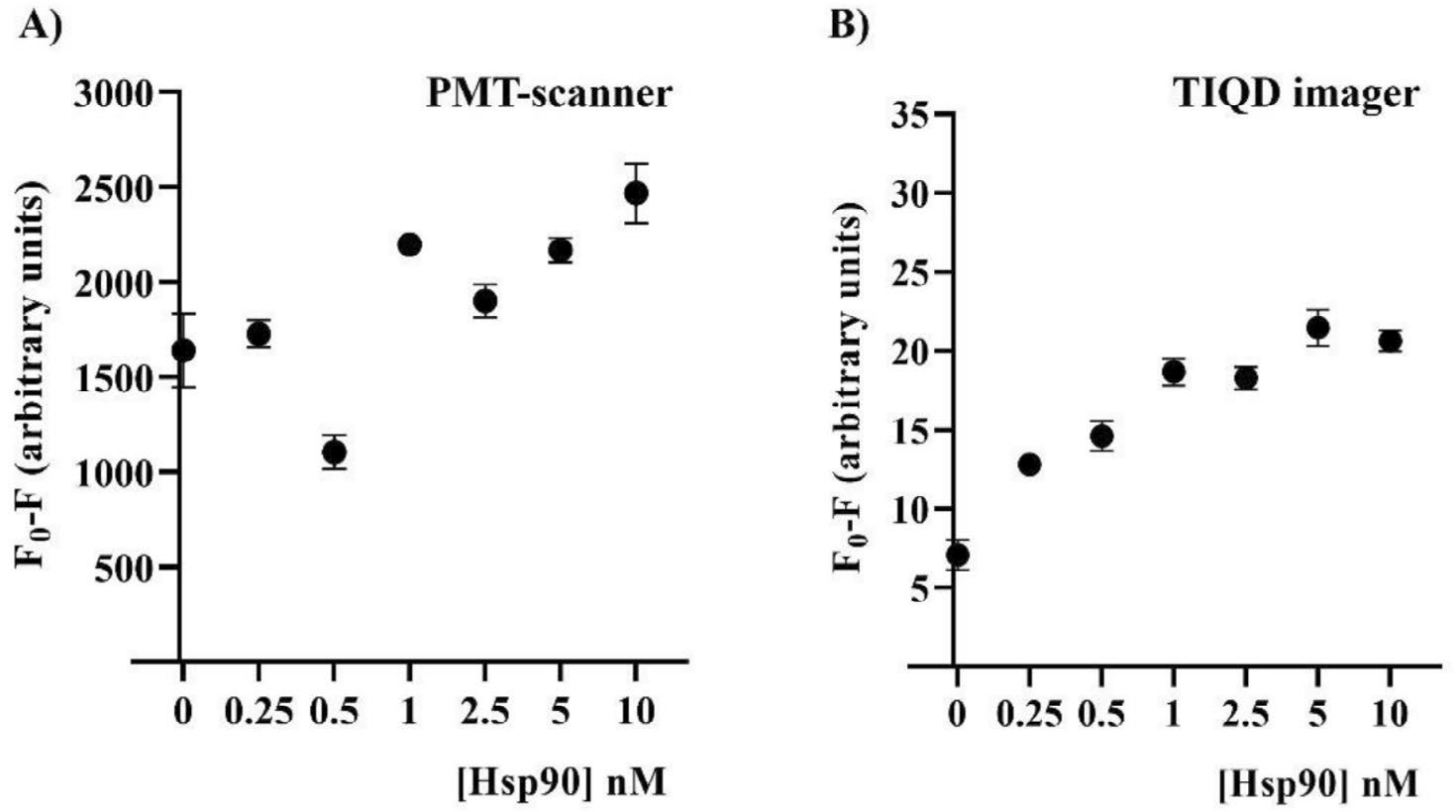
Fluorescence measurements made before and after adding blood serum spiked with His‐tag Hsp90 to the biochip and then 250 nM of Anti‐His (secondary Ab). Fluorescence masking is plotted against different concentrations of His‐tag Hsp90. (A) Shows the fluorescence signal obtained by PMT‐scanner. (B) Results from TIQD imager after adding His‐tag Hsp90 and after 250 nM secondary Ab. *F*_0_ is the fluorescence signal after QD + Ab solution is spotted on the biochip and *F* is the fluorescence signal from these spots after incubation with blood serum spiked with His‐tag Hsp90 and Anti‐His.

The overall detection range for Hsp90 was 1000–50 nM and for Hsp70 was 1000–25 nM using either the PMT‐scanner or the TIQD imager. This range is relatively high for detecting stress biomarkers within clinically significant levels in the blood or saliva of endurance athletes. As noted in the work by Lu et al., a concentration‐dependent fluorescence masking effect was observed for TRb (second protein layer) at concentrations of 1000, 10, 0.1, and 0 nM [29]. The initial fluorescence masking was induced by Hsp90 (first protein layer), with TRb attaching to Hsp90 via protein–protein interactions enhancing the masking effect. To lower the detection range of the biochip, a secondary Ab (Anti‐His) was utilized by attaching it to the His‐tag of Hsp90. A varying concentration of Hsp90 was added to the biochip following incubation with a concentration of 250 nM of the Anti‐His. This approach was intended to create the second protein layer to further mask the fluorescence of QD + Ab spots. Each concentration was tested using 10 replicates of Sav‐QD spots per Hsp90 concentration, on a single biochip. As shown in Figure 12, for both the PMT‐scanner and the TIQD imager, fluorescence masking decreases from 10 to 0.5 nM of Hsp90 for the PMT‐scanner and down to 0.25 nM for the TIQD imager. Abnormal values at 1 and 0.5 nM Hsp90 can be from biochip printing uncertainties.

### 3.5 Shelf‐Life Test

**FIGURE 13:**
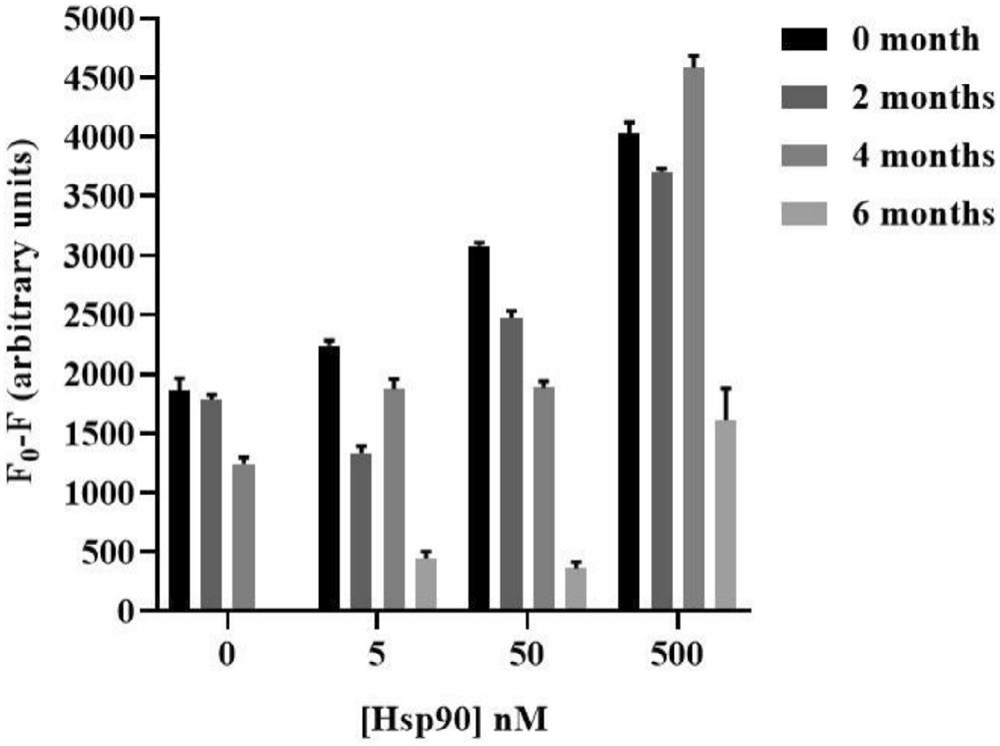
Fluorescence masking induced by different concentrations of Hsp90 showing the activity of the biochip after storing in the storage solution for up to 6 months to determine the optimum shelf life. Fluorescence measurements made by the PMT‐scanner before and after adding blood serum spiked with Hsp90. The measurements were made immediately after printing of the biochip, 2, 4, and 6 months after storage.

The transition to PoC testing is facilitated through the extension of the biochip's shelf life, enabling prolonged storage and usage. Measurements were performed on separate and single biochips after 0, 2, 4, and 6 months of storage, with 10 replicates of Sav‐QD spots per concentration of Hsp90. As depicted in Figure 13, initially, the freshly printed biochip exhibited a concentration‐dependent fluorescence masking effect by Hsp90, which was observed up to a concentration of 5 nM. Notably, this level of fluorescence masking was maintained after 2 months of storage, remaining effective up to 5 nM of Hsp90. However, after 4 months, the biochip's ability to mask fluorescence was markedly diminished, with significant effects observed only up to a concentration of 50 nM. After 6 months of storage, the fluorescence masking capacity was further compromised, being substantially reduced even at higher concentrations of Hsp90 (500 nM). These observations suggest a considerable decline in the biochip's functional activity over time. Consequently, the biochip was deemed unsuitable for practical use beyond approximately 4 months of storage due to this marked decrease in performance.

It is important to distinguish between the photostability of the Sav‐QDs and the functional activity of the biochip. While the intrinsic fluorescence intensity of the Sav‐QDs themselves remained stable over 6 months, indicating no significant photobleaching, the functional capacity of the immobilized antibodies to bind their target biomarker and induce fluorescence masking declined significantly after 4 months of storage. Therefore, the shelf life of the biochip is determined by the stability of antibody function, not the photophysical stability of the QDs. To statistically evaluate the shelf life, a one‐way ANOVA was performed on the fluorescence masking data across the different storage time points (0, 2, 4, and 6 months). The analysis yielded a *p* value of 0.0016 (*p* < 0.05) and an *r* ^2^ value of 0.8813, confirming that the observed decrease in biochip activity over time was statistically significant. This result objectively shows that the standard storage condition (4°C) is suitable for preserving biochip function for approximately 2 to 4 months.

### 3.6 Evaluation of Cross‐Reactivity With Hsp90 and Hsp70 Abs

**FIGURE 14:**
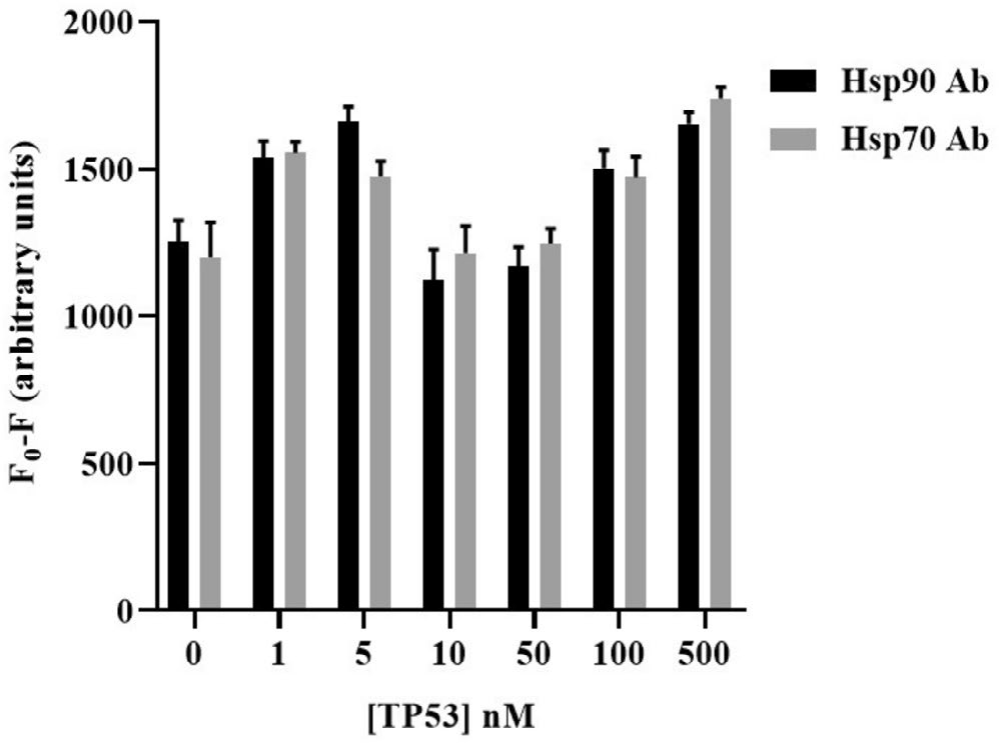
The bar graphs depict the fluorescence masking effect induced by different concentrations of TP53, examining potential cross‐reactivity between TP53 and Hsp90 or Hsp70 Abs. The fluorescence difference, or masking effect, was calculated for each TP53 concentration by subtracting the mean signal of 50 nM Sav‐QD + 500 nM Ab (Hsp90 and Hsp70) before and after incubation with TP53.

As shown in Figure 14, the values for different TP53 concentrations were close to the control (0 nM TP53). Slightly higher fluorescence differences compared to the 0 nM TP53 (control value) could be attributed to random washout effects of the buffer on the Sav‐QDs or to non‐specific binding of TP53. Each concentration was tested using 10 replicates of Sav‐QD spots per TP53 concentration on a single biochip.

**FIGURE 15:**
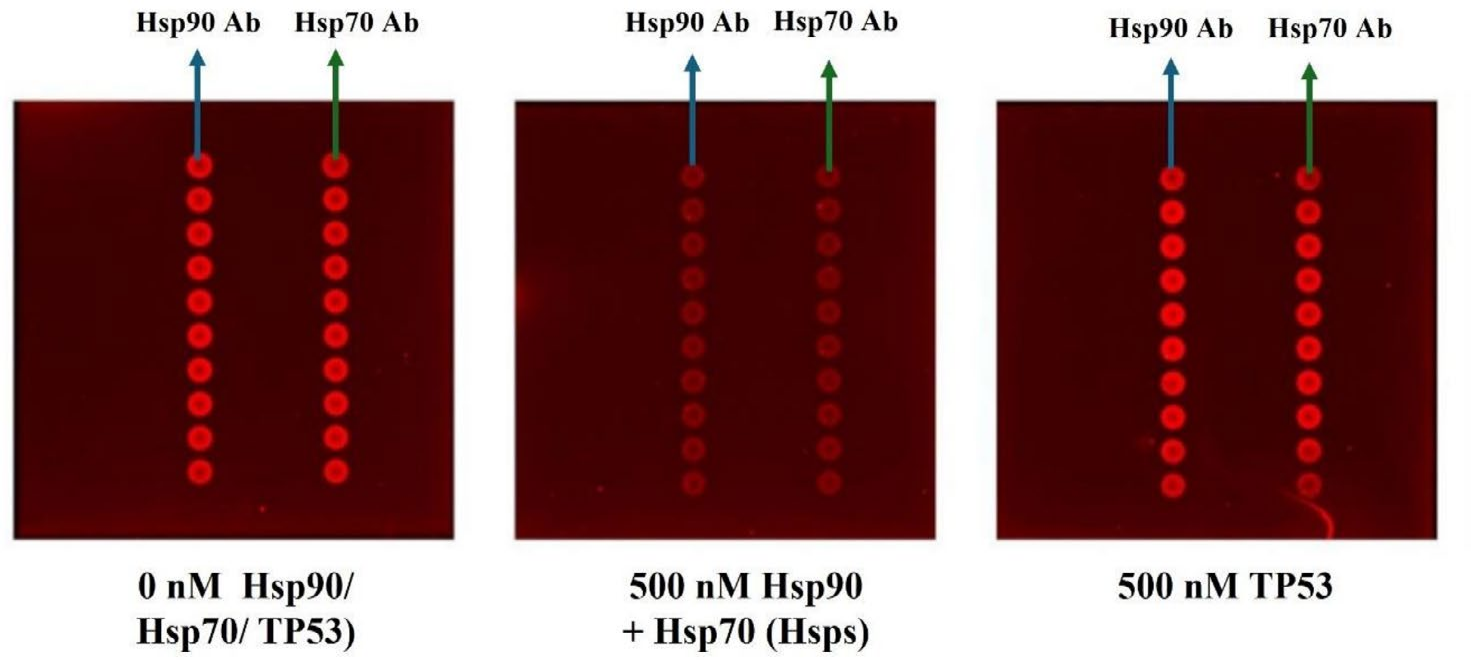
The image illustrates the fluorescence masking effect of 500 nM Hsp90 and Hsp70 together (Hsps) when bound to their respective Abs conjugated to Sav‐QDs. In contrast, TP53 showed no binding to either Hsp90 or Hsp70 Abs, resulting in no fluorescence masking. The Sav‐QD spots in the presence of TP53 remained as bright as those in the 0 nM, which contained no proteins (Hsp90, Hsp70, or TP53).

Figure 15 illustrates that the Sav‐QD + Ab spots remained as bright as without protein after the addition of 500 nM TP53. In contrast, a clear decrease in brightness was observed when 500 nM of Hsp90 + Hsp70 (Hsps) was added. Overall, no concentration‐dependent fluorescence masking was observed, suggesting that unwanted binding by random proteins like TP53 to Hsp90 or Hsp70 Abs does not take place; hence, no cross‐reactivity.

### 3.7 Biochip‐to‐Biochip Variability

**FIGURE 16:**
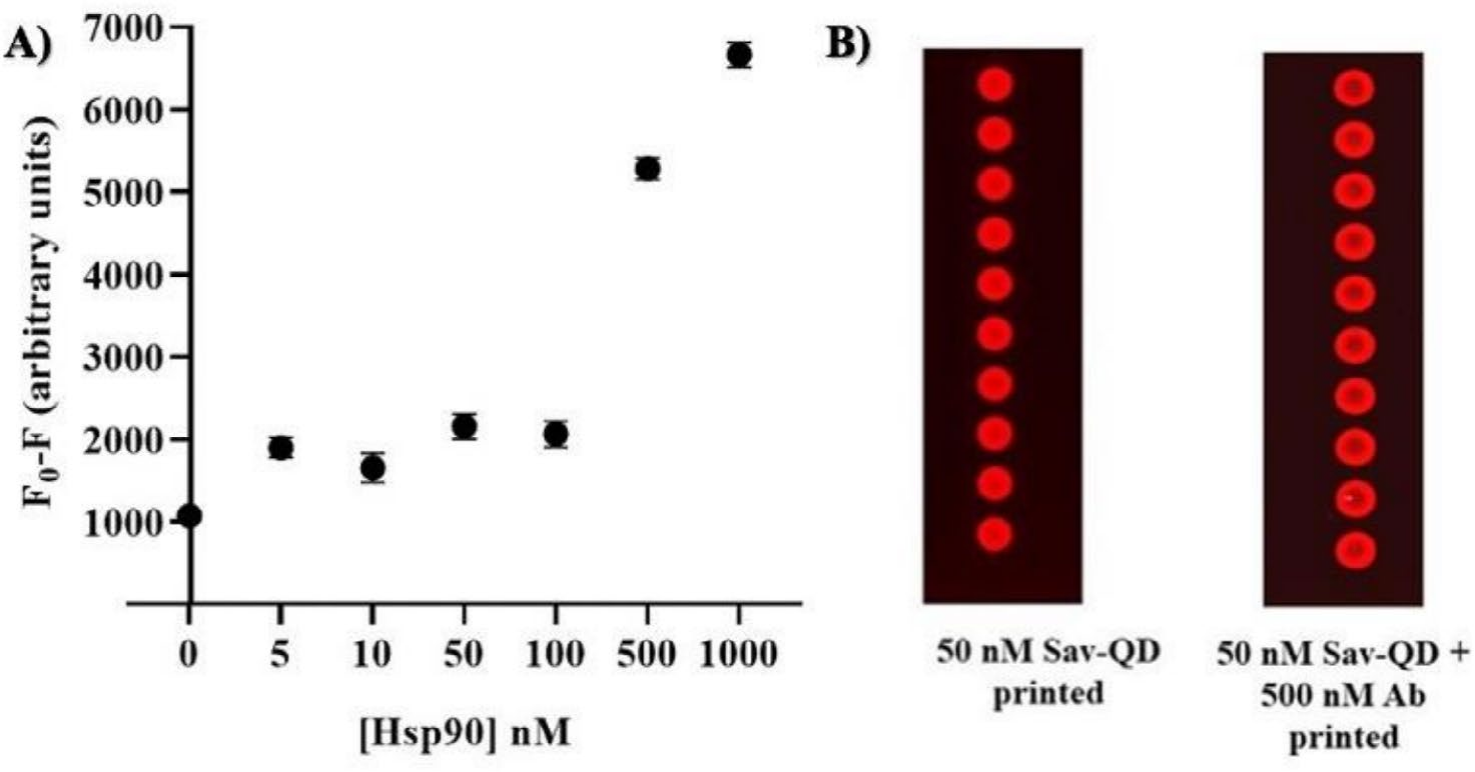
(A) Fluorescence masking induced by different concentrations of Hsp90. The graph shows average value of three different biochips. The standard error mean of all three biochips. (B) Representative images of printed spots showing the coffee ring effect: 50 nM Sav‐QD printed alone, and 50 nM Sav‐QD and 500 nM Ab printed together. A dark center is observed in each spot, indicating the coffee ring effect resulting from capillary flow‐induced particle deposition toward the droplet periphery during drying.

To assess the reproducibility, a one‐way ANOVA was performed on the fluorescence masking data (*F*_0_ − *F*) obtained from the PMT‐scanner for Hsp90 in artificial serum (Figure 16A). The analysis compared the results across three independently fabricated biochips, each tested with the full concentration range (1000, 500, 100, 50, 10, 5, and 0 nM) with 10 QD + Ab spots per concentration. The ANOVA yielded a *p* value of 0.0297, indicating a statistically significant difference between the biochips. This highlights a degree of variability in the manufacturing process that will be a focus of future optimization. Each concentration was tested using 10 replicates of Sav‐QD spots per Hsp90 concentration, on three biochips.

The graph shown in Figure 16A highlights a degree of variability in the manufacturing process. Spot uniformity was on average good across the biochips. The average spot diameter was maintained at approximately 150–200 μm with a printed volume of 0.5–1 nL per spot. Before adding test samples, every biochip was scanned to assess spot morphology. Only biochips meeting predefined quality criteria including uniform spot shape, consistent signal intensity across replicates, and absence of major printing artifacts (like aggregates of Sav‐QDs inside printed spots) were used for subsequent experiments. This pre‐scanning quality control step is widely adopted in microarray studies to minimize data variability. To address the problem of biochip‐to‐biochip variability, each biochip had its own control value. The control value was 0 nM or no stress proteins in buffer or blood serum or saliva. This control value was used as a reference for all other values on the same biochip. One of the factors for biochip‐to‐biochip variability was due to the coffee ring effect. As shown in Figure 16B, the coffee ring droplet shape was observed in printed spots of 50 nM Sav‐QD, and in the mixture of 50 nM Sav‐QD with 500 nM Ab, each exhibiting a characteristic dark center. This uneven deposition can contribute to spot‐to‐spot and biochip‐to‐biochip variability by creating heterogeneity in the local concentration of Sav‐QDs and antibodies within each spot, thereby affecting fluorescence signal consistency. Strategies to mitigate the coffee ring effect, such as optimizing solvent composition, controlling humidity during printing, or trying different biochip surfaces, will be explored in future work to improve printing uniformity and reduce overall variability. Additionally, errors originating from the TIQD imaging system might contribute to the overall signal error, although to a lesser extent. These errors can stem from drifts in the laser power or mechanical noise, which will be optimized in future versions of the system, for example, through miniaturization and the incorporation of a reference measurement point (acquired with and without proteins). This will enable normalization of laser power fluctuations across experimental sets.

### 3.8 Interpretation of Fluorescence Masking

**FIGURE 17:**
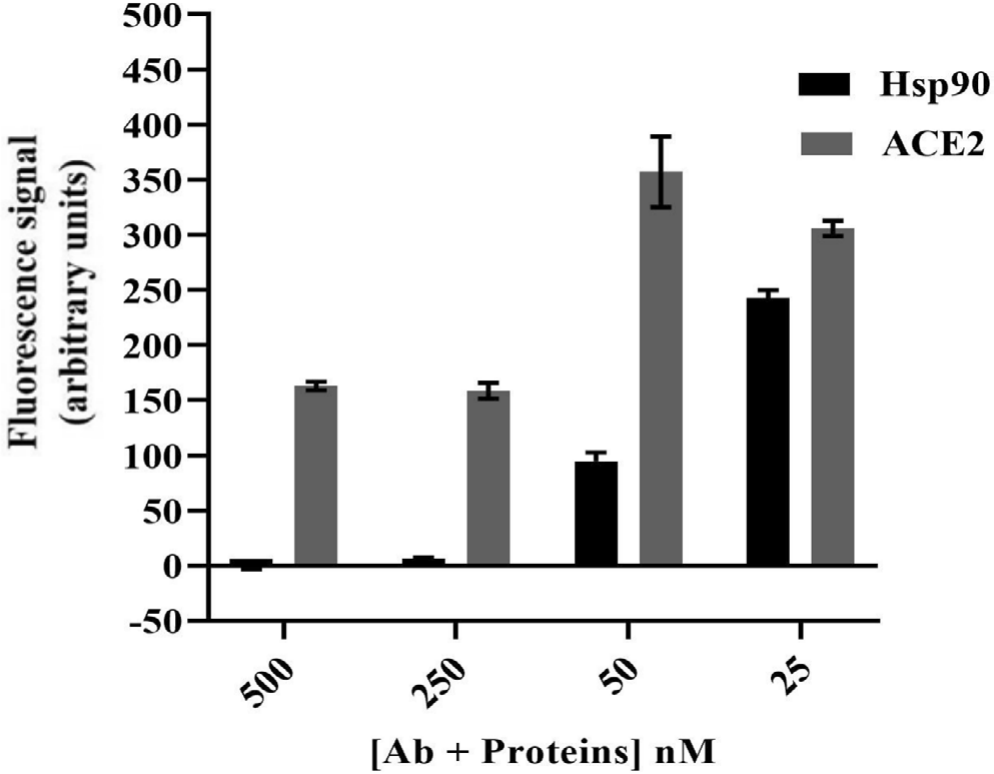
Illustration of fluorescence masking induced by different concentrations of Hsp90 and ACE2. Fluorescence signal of Sav‐QD is compared after adding different concentrations of Hsp90 and ACE2. The *X*‐axis represents the various concentrations of Ab and proteins Hsp90 and ACE2 (in nM), while the *Y*‐axis shows the corresponding fluorescence signal of 50 nM Sav‐QD, calculated as the mean from 10 Sav‐QD spots on the biochip. Error bars indicate the standard error mean of the mean fluorescence values, based on the 10 spots measured for each concentration.

To assess the size/mass‐dependent effect, fluorescence masking of Sav‐QD by Hsp90 and ACE2 was compared. Each concentration was tested using 10 replicates of Sav‐QD spots per ACE2 and Hsp90 concentration, on a single biochip. As shown in Figure 17, fluorescence masking by the larger Hsp90 dimer was more profound than that by the smaller ACE2 at each concentration. This was clearly evident as the fluorescence signal from Sav‐QD was lower in the presence of Hsp90 than in the presence of ACE2 at the same concentration. For Hsp90, complete fluorescence masking was observed at 500 and 250 nM concentrations; this was not seen for ACE2. Concentration‐dependent fluorescence masking is observed up to 25 nM Hsp90 and 50 nM ACE2. A complete loss of fluorescence at higher concentrations of Hsp90 indicates the formation of aggregates with the Sav‐QD + Ab + Hsp90 complex. Further investigations should be carried out to determine if this type of fluorescence masking is static quenching, dynamic quenching, or a combination of both.

## 4 Conclusion

The concentration‐dependent fluorescence masking observed in this work is attributed to the binding of Hsp90 spiked in blood serum to the biotinylated Hsp90 Ab of the printed QD + Ab. This trend aligns with previous findings on Hsp90 in buffer solutions [40, 41, 42]. The fluorescence masking results for both Hsp90 and Hsp70 demonstrate a similar trend. This confirms that our setup is capable of testing both stress biomarkers, Hsp90 and Hsp70, in blood serum. Notably, higher fluorescence masking was observed with Hsp90 compared to Hsp70, attributed to Hsp90 being a larger dimeric molecule (180 kDa) relative to Hsp70 (70 kDa). Consequently, this fluorescence masking concept can be employed as a relative mass detector for large proteins as well. The data presented in Figure 17 provide evidence that the fluorescence masking observed in this assay is primarily a size‐dependent, steric phenomenon. By comparing the effects of Hsp90 (~180 kDa dimer) and ACE2 (~85.9 kDa), we demonstrated that the larger protein consistently induces a greater reduction in Sav‐QD fluorescence at equivalent concentrations of the smaller protein. This size‐dependent effect supports our interpretation that the proteins, when bound via specific antibodies in close proximity to the QD surface, physically block the excitation light or emitted fluorescence, a mechanism distinct from nonspecific interactions. The complete masking may indicate the formation of higher‐order protein complexes or aggregates around the QD at higher protein concentrations. While steric blocking remains the most probable primary mechanism based on the size comparison, the data do not entirely rule out minor contributions from local refractive index changes or protein‐induced QD aggregation. Further investigations are required, which was out of the scope of the current work.

Results obtained with artificial saliva indicate that it can be utilized in the current biochip configuration for assessing stress biomarkers, though further optimization with extraction buffers and 15 min incubation time is necessary to achieve performance levels comparable to blood serum tests. Overall, the results highlight the potential of the biochip as a rapid test for stress biomarkers in saliva, potentially for home use. The simultaneous detection of Hsp90 and Hsp70 illustrates that the biochip is well suited for dual biomarkers analysis. Results for each biomarker can be obtained individually and collectively as a comprehensive stress level test. Without further measures, the system's lower detection limit stands at approximately 25 nM. In this work, we were able to reduce it to 0.25 nM by additional functionalization. In Figure 12, it is shown that the masking effect increases after adding 250 nM of secondary antibodies compared to Hsp90 alone. The addition of a second protein layer via protein–protein interactions successfully enhances the fluorescence masking effect. More cost‐effective and simpler methods to create this secondary masking protein layer, such as using a basic, bulky protein or nanoparticles that attached via non‐specific interactions, would be more suitable for a final PoC system. These large nanostructures offer a highly efficient mechanism for fluorescence blocking, potentially yielding a much greater signal attenuation per binding event. Further refinement to reach the lower pM range is required in future for stress biomarkers detection in blood serum at clinical level of sensitivity [48]. This shall be achieved by utilizing secondary nanoparticles that can be conjugated as additional binders. Approximately a 4‐month storage period for the biochip is considered adequate for testing higher concentrations, while 2 months suffices when testing lower concentrations of stress biomarkers. Optimizing storage conditions could extend the biochip's shelf life to approximately 10–12 months. With enhanced optics and microfilters, clearer images of the biochip are anticipated with a smartphone camera, illustrating concentration‐dependent fluorescence masking by stress biomarkers. Future work will also include image analysis for biomarker concentration data and the development of a miniaturized imager based on a standard smartphone, in contrast to the CMOS camera currently used, which will simplify, miniaturize, and improve system handling and user‐friendliness.

As Hsp70 and Hsp90 are upregulated in response to physiological stress, such as that induced in endurance athletes during intensive training, Hsp levels are integrally linked to the subsequent immune and inflammatory responses, which are critical components of the overall stress and recovery cycle in athletes or people recovering from surgeries [49, 50]. Notably, saliva is generally preferred over blood tests by users due to its non‐invasive nature and the absence of pain while sampling or waste disposal concerns associated with biochip. The biochip was successfully tested with artificial saliva samples spiked with Hsp90, although its efficiency was slightly lower compared to blood serum. To optimize performance, pre‐treatment of saliva samples was necessary prior to adding on the biochip. However, a significant advantage of the presented test system was the elimination of the need for additional chemical treatment during testing [51].

Future efforts will focus on the miniaturization of the TIQD imager system, facilitating its evolution into a smartphone integrated, portable, and cost‐effective device [52, 53, 54]. This will be achieved by miniaturizing individual optical imaging components through the use of micro‐optics, potentially realized via additive manufacturing technologies [55, 56, 57]. Techniques such as hot imprint replication, two‐photon polymerization, or microscope projection photolithography can be employed to create integrated light‐guiding and imaging elements, including waveguides, micro‐lens arrays, and diffractive optical elements [58, 59, 60]. In summary, the strong correlation between our TIQD imager and the PMT‐scanner demonstrates that our portable system performs reliably against established technology. A comparison with commercial lateral flow assays, which currently typically offer single‐analyte and qualitative/semi‐quantitative results, will be conducted once the system is optimized for real‐sample analysis at clinically relevant sensitivities. In the context of physical stress in sports during the training phase of athletes, circulating Hsp70 levels vary significantly depending on exercise intensity, duration, and recovery time. For athletes immediately after training, extracellular Hsp70 concentrations typically range from approximately 70–7 pM, with levels returning to baseline within 2 h post‐exercise and complete recovery after 24 h. Studies using commercially available ELISA methods have reported detectable plasma Hsp70 levels during rest and exercise stress, with elevations observed in response to acute exercise and environmental stress. These values are substantially lower than the primary detection range of the biochip, highlighting the need for signal amplification strategies to achieve the clinically meaningful detection range [61, 62]. For Hsp90 alpha, elevated serum or plasma levels have been established as diagnostic and prognostic biomarkers in several malignancies. In liver cancer and advanced gastrointestinal tumors, circulating Hsp90 alpha levels are significantly elevated compared to healthy controls. High Hsp90 alpha expression correlates with poor survival outcomes in liver cancer, kidney renal clear cell carcinoma, lung adenocarcinoma, breast cancer, and mesothelioma. The clinically relevant ranges generally fall within the low nanomolar to sub‐nanomolar range, which aligns with the detection limit achieved using our secondary antibody strategy (≈0.25 nM) [63]. While the secondary antibody amplification strategy successfully reduces the limit of detection to clinically relevant ranges (≈0.25 nM), it introduces additional steps that add complexity to the workflow and may limit the PoC applicability of this approach. Therefore, further work is required to achieve clinically significant detection ranges using alternative, simplified strategies. The future work involves the use of gold nanoparticles as fluorescence quenchers in a mixing‐and‐measuring format, wherein a general masking agent or blocker can be mixed directly with the test sample before transfer to the biochip. This approach, inspired by established principles of fluorescence quenching between quantum dots and gold nanoparticles, may enable a simplified, single‐step detection format suitable for PoC applications without compromising sensitivity [26]. Beyond gold nanoparticles, other nanomaterials have been extensively investigated as fluorescence quenchers for quantum dot‐based sensing platforms. Carbon quantum dots and graphene quantum dots have emerged as promising alternatives due to their biocompatibility, low toxicity, and efficient quenching capabilities via FRET or electron transfer mechanisms [64, 65, 66].

## Author Contributions

Anusha Kishore wrote the manuscript and was responsible for biochip printing, usage, and biomarker testing with the PMT‐scanner. Arun Mathew Varughese constructed the TIQD imager and took the optical measurements. Bernhard Roth and Carsten Zeilinger were responsible for data validation, project coordination, and management. All authors contributed to the iteration of the manuscript.

## Funding

This work was supported by the Deutsche Forschungsgemeinschaft (Grant 390833453) and the NBank (State Bank for Lower Saxony) through the incubator project MSMBest as part of the SMINT@Hannover program.

## Conflicts of Interest

The authors declare no conflicts of interest.

## Data Availability

The data that support the findings of this study are available on request from the corresponding author. The data are not publicly available due to privacy or ethical restrictions.
